# A Cross-Sectional Assessment of HIV Self-Testing Preferences and Uptake Among Key Populations in Phnom Penh, Cambodia

**DOI:** 10.9745/GHSP-D-21-00412

**Published:** 2022-06-29

**Authors:** Michael M. Cassell, Philippe Girault, Sopha Nith, Chandary Rang, Sereyvisith Sokhan, Sovannary Tuot, Vichet Kem, Pagna Dork, Aphyra Chheav, Mary Sos, Chanry Im, Sotheary Meach, Kimrun Mao, Penh Sun Ly, Vohith Khol, Sovannarith Samreth, Bora Ngauv, Vichea Ouk, Sopheap Seng, F. Stephen Wignall

**Affiliations:** a FHI 360, Hanoi, Vietnam.; b FHI 360, Phnom Penh, Cambodia.; c Khmer HIV/AIDS Nongovernmental Organization Alliance, Phnom Penh, Cambodia.; d Men's Health Cambodia, Phnom Penh, Cambodia.; e Men's Health Social Services, Phnom Penh, Cambodia.; f Cambodian Women for Peace and Development, Phnom Penh, Cambodia.; g Chhouk Sar Association Clinic, Phnom Penh, Cambodia.; h National Center for HIV/AIDS, Dermatology, and Sexually Transmitted Diseases, Phnom Penh, Cambodia.

## Abstract

**Background:**

HIV self-testing (HIVST) is recommended by the World Health Organization, but implementation remains limited. This cross-sectional study evaluated HIVST uptake among female entertainment workers (EWs), men who have sex with men (MSM), and transgender women in Phnom Penh, Cambodia, to inform national implementation.

**Methods:**

Between December 2018 and September 2019, individuals reached through community outreach or via online advertising were offered HIVST or referrals to facility-based testing. Participants opting for HIVST could choose between test kits employing oral-fluid or finger-prick-based sample collection; and between an “assisted” option in which outreach staff offered instructions and assistance and an “unassisted” option in which participants received a kit with instructions for use. A structured questionnaire was administered to facilitate descriptive statistics and tests for associations between participant characteristics and HIV testing preferences and outcomes.

**Results:**

Among 1,241 eligible individuals; 1,210 (97.5%) provided responses for analysis. Of these, 1,203 (99.4%) were recruited through outreach; 7 (0.6%) through online advertising. Among those recruited by outreach, 1,186 (98.6%) opted for assisted HIVST, and 1,065 (88.5%) opted for oral-fluid versus finger-prick testing. All individuals recruited through online advertisements opted for unassisted oral-fluid testing. Among all participants, 455 (37.6%) were MSM, 325 (26.9%) were transgender women, 430 (35.5%) were female EWs, and overall, 71.7% reported never previously testing for HIV. A total of 84 participants (6.9%) received reactive screening results and 81 (97.5%) were linked to treatment.

**Conclusion:**

Uptake of HIVST was high, and most participants preferred oral-fluid over finger-prick-based testing. Many individuals (72%) who had never previously accessed HIV testing services participated in HIVST, with high rates of reactivity.

## Abstract (precis)

Offering HIV self-testing services to key populations in Cambodia expanded HIV testing access to a large proportion of individuals with no prior testing history and resulted in high rates of new HIV case detection and subsequent linkages to HIV treatment.

## Teaser

**Key Findings:**

- All study participants opted for HIV self-testing (HIVST) over referrals to facility-based testing services, with the vast majority selecting assisted options and a preference for oral-fluid versus finger-prick-based HIV test kits.
- High rates of reactivity and subsequent links to HIV treatment among study participants—the majority of whom had no prior HIV testing history—suggest that HIVST is an empowering option for individuals who are at significant risk but are reticent to test due to perceived stigma or embarrassment.

**Key Implications:**

National HIV program implementers should:

- Expand implementation of HIVST through peer-mediated and community outreach channels, consistent with new national HIVST guidelines adopted following completion of this study.
- Advocate for and promote differentiated HIVST access options, including options for HIVST online ordering and pharmacy distribution, to help overcome barriers to HIV testing during the coronavirus disease (COVID-19) pandemic and beyond.

## INTRODUCTION

HIV self-testing (HIVST) is recommended by the World Health Organization (WHO) as a strategy to overcome the barriers many individuals face to learning their HIV status and accessing lifesaving HIV treatment and prevention services.^1^ By offering self-led and discreet testing options, HIVST can open up preferred pathways for individuals who might not otherwise access services due to concerns about conven-ience, confidentiality, stigma, and discrimination.^2^^,^^3^ A growing evidence base underscores the feasibility, reliability, safety, and acceptability of HIVST.^1^^,^^3^^–^^6^ Implementation experiences have found HIVST to be attractive to individuals who have not previously tested,^4^ and randomized controlled trial findings have demonstrated an association between access to HIVST and increased uptake and frequency of HIV testing.^7^ Studies suggest that key populations (KPs) facing elevated HIV infection risks—such as men who have sex with men (MSM), transgender women, sex workers, and people who inject drugs—often prefer oral-fluid HIVST over facility-based and blood-based testing.^5^^,^^8^^–^^13^

Nevertheless, in Cambodia and several other countries globally, historical implementation of HIVST has been constrained due to lingering local doubts about feasibility, acceptability, demand, accuracy, and safety.^3^ Until recently, Cambodia's HIV testing policy and regulatory frameworks precluded implementation of HIVST pending demonstration of a practical approach.^14^ A previous qualitative assessment supporting HIVST acceptability among transgender women, MSM, and female entertainment workers* (EWs) was conducted in 2016. However, since HIVST was not available in Cambodia at the time, participants' responses were unlikely to have been based on actual experiences with HIVST.^15^

Implementation of HIV self-testing has been constrained due to lingering doubts about feasibility, acceptability, demand, accuracy, and safety.

The Linkages across the Continuum of HIV Services for Key Populations Affected by HIV (LINKAGES) project—funded by the U.S. Agency for International Development through the U.S. President's Emergency Plan for AIDS Relief—focused on the development of more effective and sustainable systems to reach, test, treat, and retain KPs in HIV prevention, treatment, care, and support services. In 2018, LINKAGES to-gether with the National Center for HIV/AIDS, Dermatology, and Sexually Transmitted Diseases in Cambodia and other development partners proposed an implementation study to explore the uptake of HIVST in Cambodia with a vision to inform prospective policy changes supporting scale-up locally.

In particular, the study assessed participant preferences in choosing between (1) assisted HIVST—in which trained peer outreach staff provided oral instructions before and during the procedure and assistance as requested in conducting the test and/or interpreting the result; (2) unassisted HIVST—in which participants were simply provided with an HIVST kit that included detailed instructions for conducting the test and interpreting the result; or (3) referral to facility-based testing following the national testing algorithm.^1^ Among those who selected HIVST, the study also assessed participant preferences in choosing between the use of oral-fluid-based (OraQuick) and blood-based (Alere Determine) kits.

**Figure:**
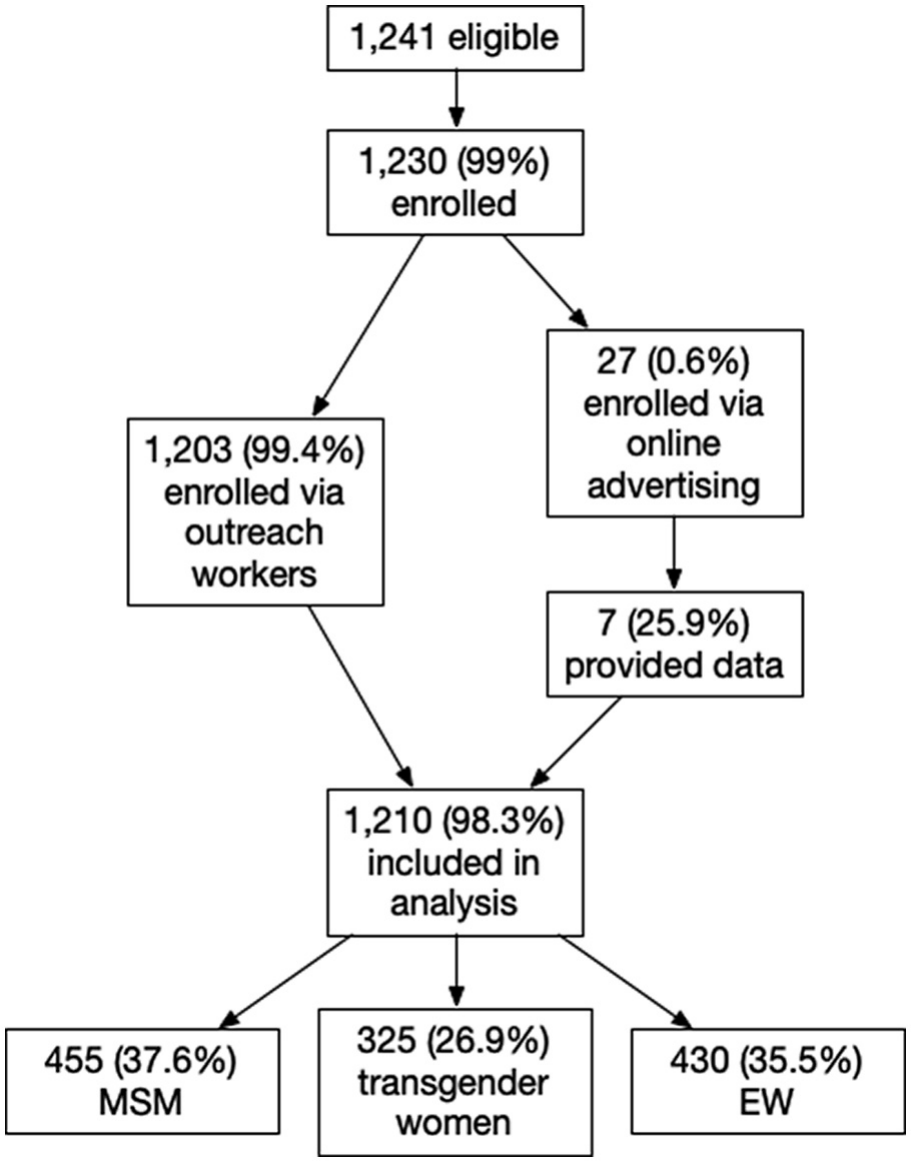
A woman performing HIV self-testing as part of a community worker's (not pictured) outreach efforts in Cambodia. Credit: © 2021 Sor Voleakhena/Cambodian Women for Peace and Development.

## METHODS

### Study Design and Setting

Between December 2018 and September 2019, the LINKAGES project collaborated with the National Center for HIV/AIDS, Dermatology, and Sexually Transmitted Diseases, the Khmer HIV/AIDS Nongovernmental Organization Alliance, and KP-led community-based organization (CBO) partners Cambodian Women for Peace and Development, Men's Health Social Services, and Men's Health Cambodia to conduct a cross-sectional study using quantitative methods in Phnom Penh.

The primary study objective was to measure the uptake of HIV oral-fluid (OraQuick Rapid HIV-1/2 Antibody Test) and blood-based (Alere Determine HIV–1/2 Ag/Ab Combo) finger-prick testing among KPs through the implementation of 3 modalities: (1) unassisted HIVST, (2) assisted HIVST, and (3) referral to HIV testing and counseling services.

The CBO partners had longstanding experience implementing HIV prevention interventions and other services for KPs in Phnom Penh through KP peer staff and were involved in reviewing tools used for the study, recruiting and enrolling participants, collecting data, and providing services re-lated to the study. Before the study launched, CBO peer outreach workers were trained on protocol-specified study procedures, including participant eligibility screening, informed consent, enrollment, data collection tools, and the step-by-step process of offering the different options for HIV testing and follow-up support according to participant preferences. The outreach workers were also trained in ethical conduct of research. LINKAGES staff provided supervision and were always available to respond to questions or concerns throughout study implementation. A group of 5 clinical service providers, representing a mix of public sector and nongovernmental sites in Phnom Penh, served as the referral network for the study, offering confirmatory HIV testing, treatment, and other services to participants. One of these sites, the Chhouk Sar Association Clinic, provides services specifically tailored to the preferences and needs of KPs.

### Study Populations

Participants in this study were female EWs, transgender women, and MSM in Phnom Penh. Eligibility criteria for enrollment included being a Cambodian citizen aged 15 years or older; being a member of a KP group; having HIV-negative or unknown status; self-reporting anal sex and/or oral sex with at least 1 male sexual partner in the past 12 months (this eligibility requirement was waived for EWs); and being willing to provide contact information (telephone number/social media username) and be contacted by study staff to allow follow-up. People who had already initiated HIV antiretroviral therapy (ART) or had taken HIV pre- or post-exposure prophylaxis in the past 3 months were not eligible. Membership in a KP group was assessed by asking participants questions about their gender identity, sex at birth, and risk behaviors. Individuals who identified as transgender women (sreysos in Khmer) or as women who were not born with female genitalia were classified as transgender women for the purposes of enrollment and analysis. Individuals who identified as male but reported anal or oral sex in the past year with at least 1 male partner were classified as MSM. Individuals who identified as females born with female genitalia, and who reported transactional or casual vaginal, anal, or oral sex with at least 1 male sexual partner in the past year, were classified as EW.

### Sampling

The sampling frame comprised female EWs, transgender women, and MSM reached either through routine face-to-face or online outreach activities of the CBO partners or by virtue of their responses to targeted online advertisements posted on popular social media channels in Cambodia. Between December 2018 and September 2019, all individuals reached during community-based and online outreach activities or who responded to the social media advertisements who met the eligibility criteria were offered participation in the study through a “take-all” approach. Peer workers from the different KP groups were engaged in outreach-based recruitment to help facilitate population-relevant sampling.

### Sample Size

Uptake of HIVST was quantified for the purposes of the study as the proportion of participants opting for HIVST as opposed to referrals to traditional facility-based HIV testing services. We calculated these proportions as the number of enrolled individuals who selected HIVST options divided by the total number of enrolled individuals, and the number of enrolled individuals who selected traditional facility-based HIV testing services divided by the total number of enrolled individuals. Accordingly, the primary endpoint used for calculating the sample size was the proportion of participants who had been tested and who received their HIV test results through assisted HIVST, unassisted HIVST, or referral. Applying the formula for a 2-sided confidence interval (CI) for one proportion, a sample size of 366 participants was calculated for each targeted KP group that would produce a 2-sided 95% CI with a half-width equal to 0.05 given a sample proportion for the primary endpoint of 0.60. This relatively high sample proportion was selected based on evidence of high HIVST acceptability in other studies.^1^^,^^3^^–^^6^ However, the study team inflated the sample size to 430 participants to accommodate 15% loss to follow-up and potential data entry errors.

### HIVST Kits

The OraQuick HIV 1/2 Rapid Antibody Test (OraSure Technologies Inc., Thailand) and Alere Determine HIV–1/2 Ag/Ab Combo (Abbott, Korea) were selected as the oral-fluid and blood-based test kits, respectively, for this study.

The OraQuick test is a qualitative, in vitro immunoassay. It detects antibodies to HIV type 1 (HIV-1) and type 2 (HIV-2) in human oral fluid, whole blood, serum, or plasma. The assay is read visually and is intended for the detection of such antibodies from individuals infected by HIV-1 or HIV-2. The OraQuick test was prequalified by the WHO in 2016, and per the WHO report, it has a high reliability in a laboratory setting when compared with results of serum/plasma-based specimens: sensitivity of 99.1% (95% CI=97.8%, 99.8%) and specificity of 99.8% (95% CI=99.2%, 100%).

The Alere Determine HIV–1/2 Ag/Ab Combo is also a qualitative, in vitro immunoassay for the simultaneous detection of HIV-1 p24 antigen and antibodies to both HIV-1 and HIV-2 in human serum, plasma, capillary (finger-prick) whole blood, or venipuncture (venous) whole blood. The Alere test is also WHO prequalified,^16^ and the manufacturer reports clinical sensitivity above 99.9% and specificity above 99.6% for all sample types.^17^

### Key Procedures

Participants were recruited through 1 of 3 approaches: (1) face-to-face peer outreach by KP CBO staff, (2) online peer outreach conducted by KP CBO staff, or (3) online advertising linked to a web-based registration platform. In the face-to-face strategy, outreach workers approached potential participants as part of their routine engagement activities in physical locations including “hot spots” such as bars and entertainment venues in which KP members often meet sexual partners, and through hosting venue-based educational events in these settings. In the online strategy, outreach workers initiated contact with potential participants as part of their routine engagement on social media such as Facebook and geosocial networking applications such as Hornet, Grindr, and Jack'd, and used a standardized recruitment script. All study procedures were conducted in a quiet and private location for face-to-face interactions and through personal telephone calls for individuals enrolled via online activities. For the online advertising recruitment strategy, advertisements were placed on social media and networking applications—known to be popular with KPs from a prior information and communications technology survey—that directed individuals with interest in the study to an online registration platform on which they could self-register, provide informed consent, self-screen for eligibility for HIVST, and select their preferred option for accessing testing including HIVST kit delivery. As detailed in the study results, there was an attempted suicide 2 months before study completion by a participant who was recruited via website and who did not opt for online counseling or assistance. In consultation with the Cambodia Ministry of Health, a decision was made to suspend all forms of recruitment that did not incorporate provision of face-to-face counseling for the outstanding duration of the study.

**Figure:**
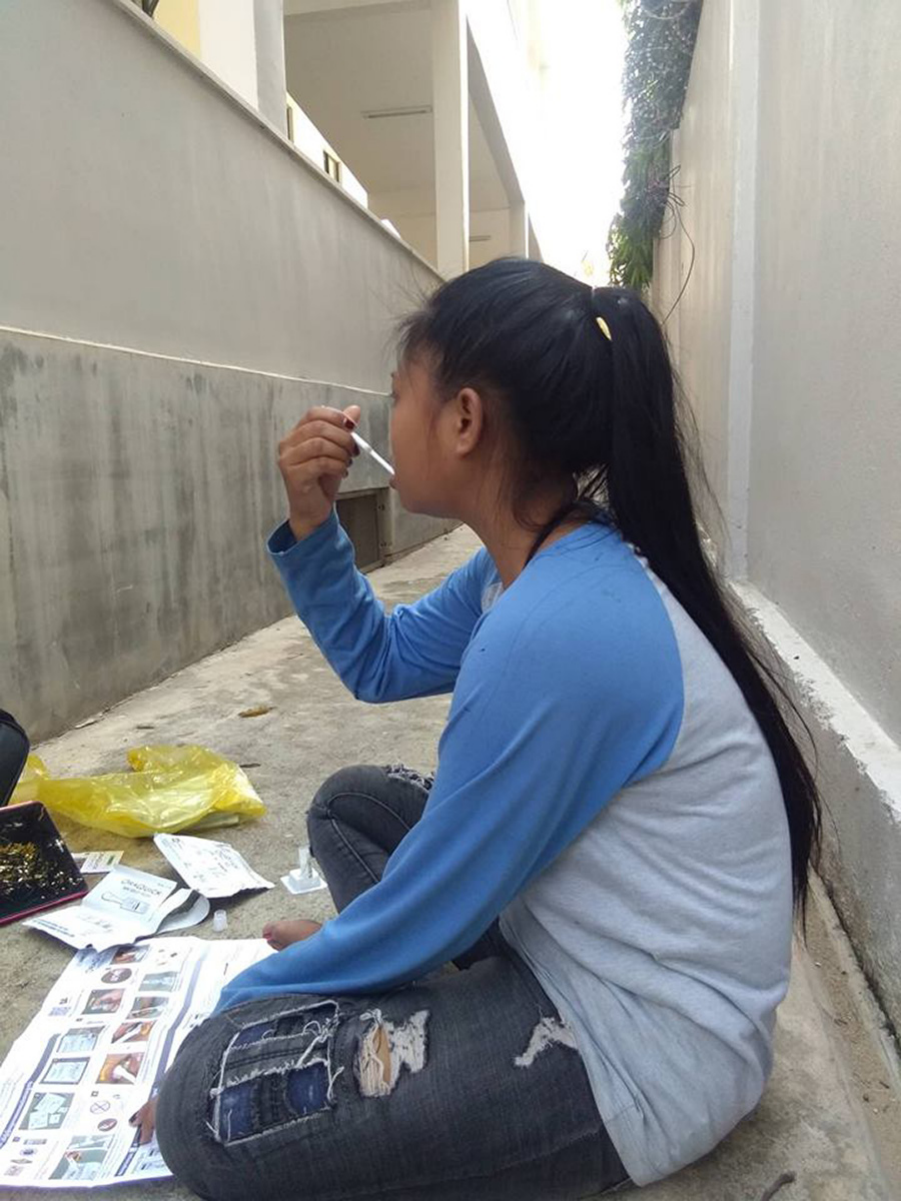
An example of HIV self-testing promotional materials that are currently used by national HIV program partners in Cambodia. Credit: © 2021 Courtesy of the Cambodia National Center for HIV/AIDS, Dermatology, and Sexually Transmitted Diseases

All eligible individuals were provided a brief explanation of the study including key procedures and were offered a choice of 3 HIV testing options: (1) unassisted HIVST, (2) assisted HIVST, or (3) routine referral to a facility for blood-based test conducted according to the national HIV testing algorithm. Individuals who selected either of the HIVST options could also opt for either oral-fluid or capillary blood-based method, using either the OraQuick or the Alere Determine Combo kit, respectively. All testing was provided free of charge. The instructions for use of the HIVST kits were developed by or in partnership with the respective manufacturer and were translated into Khmer and pretested with volunteer KP community members by the LINKAGES staff.^†^

Participants who opted for unassisted HIVST could receive kits directly from the peer outreach worker in the community, at a CBO facility, or via express courier delivery. The unassisted HIVST kits provided by the study team included the following: (1) a manufacturer's insert with pictorials and text; (2) a unique identifier code to access secure pages on the study website to view a video in Khmer language with step-by-step instructions for HIVST, to enter the result of the HIVST, and to provide answers to an online questionnaire; and (3) a referral card with contacts (e.g., the telephone number or username of a social media account) of trained outreach workers for support and assistance and selected health facilities for confirmatory HIV testing, if needed.

Participants who selected assisted HIVST were provided an HIVST kit by a trained outreach worker along with oral instructions before and during the procedure, and, when requested, assistance in conducting the test and/or interpreting the result. Those who selected assisted HIVST were invited to conduct the test at a confidential venue chosen by the KP individual and outreach worker in the proximity of the recruitment site.

Collaborating CBO staff systematically and privately followed up with participants via telephone or social media app to support linking to relevant services, monitor adverse events within 48 hours after unassisted or assisted HIVST, and, for those who opted for unassisted HIVST, to document that the test had been received and conducted. Pre-exposure prophylaxis for HIV prevention was not yet available in Cambodia during the study implementation period, but participants with nonreactive HIV test results were offered risk-reduction counseling, condoms, and support for access to other available services consistent with the standard prevention packages offered to KPs by local outreach workers and organizations. Individuals with reactive results or who were unable to interpret their result were offered assisted navigation to facility-based HIV testing services for confirmatory testing using the national HIV algorithm. Participants who were confirmed HIV positive were systematically enrolled in clinical services for ART initiation. All HIV confirmatory testing and treatment services were provided free of charge and according to standard procedures under the national HIV program.

### Data Collection Tools

The study team developed a structured questionnaire in Khmer language and uploaded it to a secure website. This allowed all participants to self-administer the survey confidentially by either visiting the site or by using the ODK application on the outreach worker's mobile device. The questionnaire included closed-ended items and Likert-type scales on the following topics: sociodemographic characteristics, sexual behavior in the past 3 months, and health-seeking behavior for HIV testing in the past year. For individuals who opted for assisted HIVST, testing results were recorded directly on the study case report form by outreach staff. Individuals who selected unassisted HIVST were asked to enter their own testing results on a secure website.

A structured case report form for adverse events was administered either via telephone or in person to participants 48 hours after their participation in HIVST. The form included questions on major signs or behaviors related to emotional and cognitive stress and suicidal ideation, attempts of suicide, non-suicidal self-injury, alcohol or drug binging, and experience of social harm. The participants were asked to report the occurrence of these signs and behaviors in the past 12 months before the HIVST procedure and after obtaining the HIVST results.

All electronic files, questionnaires, case report forms, expanded notes, transcripts, and forms were stored securely without any personal identifiers.

### Data Management and Analysis

Data from the study website were exported into Excel 2016 files, and these Excel files were imported and then appended into a single matrix using Stata version 15 (StataCorp). The data from case reporting forms (e.g., test results and adverse events) were also converted into Stata files and merged with the single matrix using the unique study identification code of the participants. The final dataset was then created and cleaned to correct discrepancies and minimize missing values by using the participants' files stored at each study site. In instances in which data were identified as missing or incomplete, members of the study team attempted to contact participants to secure the additional information. The analyses conducted to generate the study results were performed with Stata 15 or with Wizard Pro version 1.9 (Evan Miller).

The study team conducted basic descriptive statistics (proportions and measures of central tendency) on key outcome, sociodemographic, risk, and other variables, and used Chi-square tests to assess whether the values for these variables were evenly distributed among participants in each of the KP groups and among the total number of participants. For the secondary outcomes, bivariate analyses were conducted—typically employing Chi-square tests or Fisher's exact tests—to assess the independence of key outcome variables such as choice of testing approach and HIV test result from participant sociodemographic, risk behavior, and testing history data. For the bivariate analyses, the Fisher's exact or, when appropriate, the Fisher-Freeman-Halton test was employed instead of Chi-square in cases in which there were 5 or fewer observations associated with specific variable values, given issues with the reliability of Chi-square results with small sample sizes.^18^ The Fisher-Freeman-Halton test is a generalization of Fisher's exact test that gives exact *P* values when comparing distributions of more than 2 independent categories between 2 groups.^19^

The occurrence of each adverse event related to HIVST was coded as “true” when the respondent reported the manifestation of specific signs, behaviors, or social harm after undergoing HIVST but not in the past 12 months.

### Ethical Considerations

The protocol and tools were approved by Cambodia's National Ethics Committee for Health and Research and FHI 360's Protection of Human Subjects Committee in the United States of America. Before data and specimen collection, eligible participants gave their informed verbal consent. All participants who were confirmed HIV positive with the national HIV testing algorithm were referred to clinical services for ART initiation at no cost.

## RESULTS

### Enrollment

A total of 1,241 individuals reached were eligible for the study, and 1,230 (99%) were enrolled—1,203 (99.4%) through outreach worker contact and another 27 (0.6%) through online advertising recruitment. Among those enrolled through outreach worker contact, 1,034 were engaged through face-to-face recruitment (290 MSM, 315 transgender women, and 429 female EWs), and 169 through online recruitment (160 MSM, 8 transgender women, and 1 female EW). A total of 187 individuals responded to online advertisements to receive HIVST kits, but only 33 met eligibility criteria, 27 enrolled, and only 7 (5 MSM and 2 transgender women) provided HIVST results. The 20 individuals who enrolled in response to the online advertisements but did not provide online data were excluded from the analysis. As a result, the total sample size used for the quantitative analysis was 1,210 (455 MSM, 325 transgender women, and 430 female EWs) (Figure).

**FIGURE:**
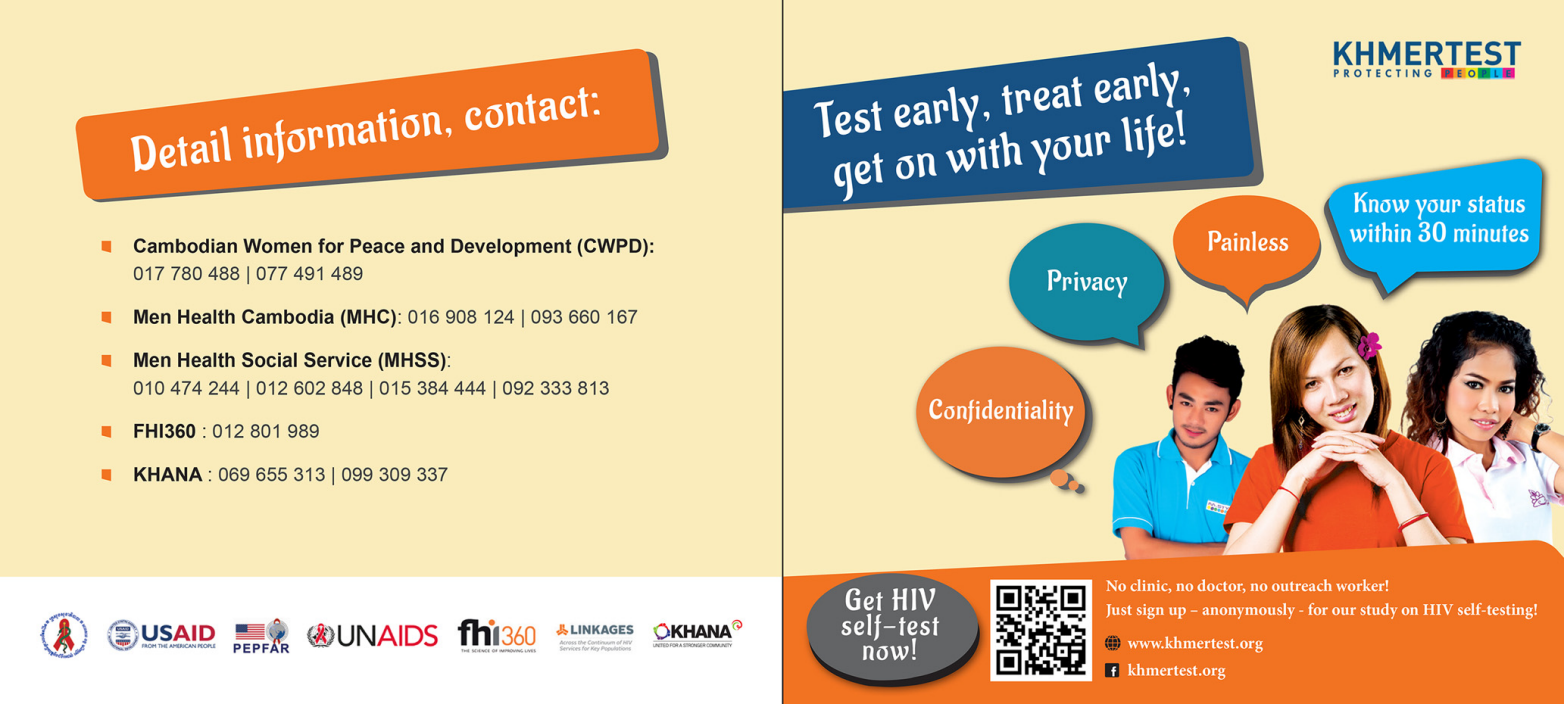
Summary of Recruitment and Enrollment Results, HIV Self-Testing Study, Phnom Penh, Cambodia Abbreviations: MSM, men who have sex with men; EW, female entertainment workers.

### Key Sociodemographic Characteristics

Table 1 summarizes the recruitment channels and key sociodemographic characteristics of the 455 MSM, 325 transgender women, and 430 female EWs enrolled in the study. The mean age of MSM was 27.5 years (median 27, standard deviation [SD]=4.96, range 18 to 49 years), the mean age for transgender women was 26.9 years (median 26, SD = 5.76, range from 18 to 55 years), and the mean age for female EWs was 25.6 years (median 25, SD = 5.74, range 17 to 45 years). More than one-third (39%) of all participants were between the age 15 and 24 years. Most MSM and transgender women reported being single (93% and 93.2%, respectively), while most female EWs were married (55%) and only 36% reported being single. Overall, about one-third of participants (35%) reported having completed high school or a higher level of education, but the proportion of EWs with this level of education was substantially lower (58% MSM, 43% transgender women, 1% female EWs). Less than 5% of participants reported being unemployed, but this proportion also varied by risk group (7.3% MSM, 6.2% transgender women, and 0% female EWs).

**TABLE 1:** Recruitment Channels and Key Sociodemographic Characteristics of Participants in Key Population Groups, HIV Self-Testing Study, Phnom Penh, Cambodia

	Men Who HaveSex With Men		TransgenderWomen		EntertainmentWorkers		Total	
	No. (%)(N=455)	*P* Value	No. (%)(N=325)	*P* Value	No. (%)(N=430)	*P* Value	No. (%)(N=1,210)	*P* Value
Mode of recruitment		<.001		<.001		<.001		<.001
Outreach worker face-to-face	290 (63.7)		315 (96.9)		429 (99.8)		1,034 (85.5)	
Outreach worker online	160 (35.2)		8 (2.5)		1 (0.2)		169 (14.0)	
Online advertising	5 (1.1)		2 (0.6)		0		7 (0.6)	
Age group		<.001		<.001		<.001		<.001
<18 years	0		0		2 (0.5)		2 (0.2)	
18–24 years	132 (29.0)		124 (38.2)		207 (48.1)		463 (38.3)	
25–29 years	189 (41.5)		112 (34.5)		118 (27.4)		419 (34.6)	
30–34 years	93 (20.4)		61 (18.8)		64 (14.9)		218 (18.0)	
35+ years	41 (9.0)		28 (8.6)		39 (9.1)		108 (8.9)	
Level of education completed		<.001		<.001		<.001		<.001
No schooling	6 (1.3)		7 (2.2)		143 (33.3)		156 (12.9)	
Primary	44 (9.7)		61 (18.8)		205 (47.7)		310 (25.6)	
Secondary	130 (28.6)		114 (35.1)		77 (17.9)		321 (26.5)	
High school	189 (41.5)		98 (30.2)		5 (1.2)		292 (24.1)	
At least 1 university degree	75 (16.5)		42 (12.9)		0		117 (9.7)	
No answer	11 (2.4)		3 (0.9)		0		14 (1.2)	
Current occupation		<.001		<.001		<.001		<.001
Unemployed	33 (7.3)		20 (6.2)		0		53 (4.4)	
Student	56 (12.3)		12 (3.7)		0		68 (5.6)	
Civil servant	15 (3.3)		8 (2.5)		0		23 (1.9)	
Self employed/freelance	165 (36.3)		81 (24.9)		2 (0.4)		248 (20.5)	
Employee outside government	95 (20.9)		52 (16.0)		0		147 (12.1)	
Hotspot worker^a^	66 (14.5)		134 (41.2)		423 (98.4)		623 (51.5)	
Other	13 (2.8)		13 (4.0)		5 (1.2)		31 (2.6)	
No answer	12 (2.6)		5 (1.5)		0		17 (1.4)	
Current marital status		<.001		<.001		<.001		<.001
Married to a man	11 (2.4)		20 (6.2)		235 (54.7)		266 (22.0)	
Married to a woman	14 (3.1)		1 (0.3)		1 (0.2)		16 (1.3)	
Divorced/separated	3 (0.7)		0		34 (7.9)		37 (3.1)	
Widowed	2 (0.4)		1 (0.3)		5 (1.2)		8 (0.7)	
Single	423 (93.0)		303 (93.2)		155 (36.0)		881 (72.8)	
No answer	2 (0.4)		0		0		2 (0.2)	

a Derived by combining the categories “massager,” “beer promoter,” “escort girl,” “hotspot manager,” and “salon worker” to mitigate risks of inadvertent disclosure of participant identity given that some cells only contained 1 or 2 individuals.

### Behavioral Characteristics, HIV Testing, and Interventions

Table 2 summarizes the self-reported risk behaviors of participants, stratified by KP typology. Overall, a majority reported using a condom during their last sexual act with a male partner (59%), but this proportion varied by KP typology (35% MSM, 49% transgender women, 93% female EWs). Among MSM, almost half (47%) reported an always insertive role during anal sex in the last 3 months, while 37% reported both insertive and receptive roles, and 15% reported exclusively a receptive role. Conversely, among transgender women, 82% reported exclusively a receptive role during anal sex during the last 3 months, while 15% reported both insertive and receptive roles, and only about 2% reported exclusively an insertive role. More than half of participants (56%) reported receiving money or goods in exchange for sex in the past 3 months from a male or transgender partner, again with considerable variation by KP typology (22% MSM, 46% transgender women, 99% female EWs). Conversely, the majority (73.3%) of MSM and transgender women reported not giving money or goods in exchange for sex in the past 3 months (70% MSM, 79% transgender women).

**TABLE 2.** Key Behavioral Characteristics of Participants in Key Population Groups, HIV Self-Testing Study, Phnom Penh, Cambodia

	Men Who HaveSex With Men		TransgenderWomen		EntertainmentWorkers		Total	
	No. (%)(N=455)	*P* Value	No. (%)(N=325)	*P* Value	No. (%)(N=325)	*P* Value	No. (%)(N=1,210)	*P* Value
Condom use at last sex with casual male partner^a^		<.001		.739		<.001		<.001
No	294 (64.6)		165 (50.8)		28 (6.5)		487 (40.2)	
Yes	159 (34.9)		159 (48.9)		401 (93.3)		719 (59.4)	
No sex in the past 3 months	0		0		1 (0.2)		1 (0.1)	
No answer	2 (0.4)		1 (0.3)		0		3 (0.2)	
Total	455 (100)		325 (100)		430 (100)		1,210 (100)	
Frequency of condom use during the past 3 months^a^		<.001		<.001		<.001		<.001
Never	127 (27.9)		75 (23.1)		19 (4.4)		221 (18.3)	
Sometimes	243 (53.4)		183 (56.3)		319 (74.2)		745 (61.6)	
Most of the time	77 (16.9)		46 (14.2)		90 (20.9)		213 (17.6)	
Always	8 (1.8)		20 (6.2)		2 (0.5)		30 (2.5)	
No answer	0		1 (0.3)		0		1 (0.1)	
Total	455 (100)		325 (100)		430 (100)		1,210 (100)	
Role during anal sex in the past three months		<.001		<.001		NA		<.001
Always insertive	214 (47.0)		6 (1.8)		NA		220 (28.2)	
Always receptive	68 (14.9)		266 (81.8)		NA		334 (42.8)	
Both receptive and insertive	168 (36.9)		50 (15.4)		NA		218 (27.9)	
No anal sex in the past 3 months	0		1 (0.3)		NA		1 (0.1)	
No answer	5 (1.1)		2 (0.6)		NA		7 (0.9)	
Total	455 (100)		325 (100)		NA		780 (100)	
Received money or goods for sex from a male or TG partner^b^		<.001		<.001		<.001		<.001
No	347 (76.3)		175 (53.8)		5 (1.2)		527 (43.6)	
Yes	102 (22.4)		149 (45.8)		425 (98.8)		676 (55.9)	
No answer	6 (1.3)		1 (0.3)		0		7 (0.6)	
Total	455 (100)		325 (100)		430 (100)		1,210 (100)	
Gave money or goods for sex to a male or TG partner^a^		<.001		<.001		NA		<.001
No	317 (69.7)		257 (79.1)		NA		574 (73.6)	
Yes	128 (28.1)		66 (20.3)		NA		194 (24.9)	
No answer	10 (2.2)		2 (0.6)		NA		12 (1.5)	
Total	455 (100)		325 (100)		NA		780 (100)	

Abbreviations: NA, not asked; TG, transgender.

a Last anal sex for men who have sex with men and TG and last vaginal sex for entertainment workers.

b In the past 3 months.

Table 3 summarizes the prior HIV testing experience and exposure to HIV interventions reported by participants. In all, 72% of participants reported never previously having had an HIV test. Eighty-four percent of female EWs were first-time testers, followed by MSM (71%) and transgender women (57%). Despite the majority having been recruited through routine community outreach activities, a low proportion (12.4%) of individuals across all KP groups reported having prior exposure to face-to-face or small group discussion activities focused on HIV prevention, care, or treatment in the past year. This proportion varied by KP typology but remained low across all typologies (13% MSM, 24% transgender women, 3% EWs). The study was conducted in the context of routine outreach, but the large number of KP members and limited number of outreach staff may account for limited prior exposure.

**TABLE 3.** Participant History of HIV Testing and Exposure to Interventions, HIV Self-Testing Study, Phnom Penh, Cambodia

	Men Who HaveSex With Men		TransgenderWomen		EntertainmentWorkers		Total	
	No. (%)(N=455)	*P* Value	No. (%)(N=325)	*P* Value	No. (%)(N=430)	*P* Value	No. (%)(N=1,210)	*P* Value
Last HIV test done		<.001		<.001		<.001		<.001
Never tested	325 (71.4)		186 (57.2)		360 (83.7)		871 (72.0)	
In past 3 months	0		0		1 (0.2)		1 (0.1)	
Between 3 and 6 months	3 (0.7)		3 (0.9)		8 (1.9)		14 (1.2)	
Between 6 and 12 months	33 (7.3)		32 (9.8)		47 (10.9)		112 (9.3)	
More than 12 months	86 (18.9)		102 (31.4)		13 (3.0)		201 (16.6)	
No answer	8 (1.8)		2 (0.6)		1 (0.2)		11 (0.9)	
First-time tester		<.001		.007		<.001		<.001
Yes	322 (70.8)		185 (56.9)		360 (83.7)		867 (71.7)	
No	125 (27.5)		137 (42.2)		69 (16.0)		331 (27.4)	
No answer	8 (1.8)		3 (0.9)		1 (0.2)		12 (1.0)	
Exposure to tailored HIV interventions in the past 12 months		<.001		<.001		<.001		<.001
Yes	58 (12.7)		79 (24.3)		13 (3.0)		150 (12.4)	
No	381 (83.7)		242 (74.5)		417 (97.0)		1,040 (86.0)	
No answer	16 (3.5)		4 (1.2)		0		20 (1.7)	

### Uptake of HIV Self-Testing

All 1,210 enrolled participants who provided data opted to participate in HIVST rather than a referral to clinic-based testing services, indicating exceptionally high uptake of HIVST as defined in the study. Almost all participants (98%) selected assisted HIVST, and the vast majority (88.6%) opted for the use of an oral-fluid-based test kit. Table 4 summarizes the uptake of the different HIVST approaches, kit types, and test kit delivery options. We found uneven distributions of both HIVST kit type selected and delivery option, by KP typology. Only 13 participants chose delivery via courier service, and 7 (53.8%) of these enrolled via online advertising and opted for unassisted HIVST. Among the 1,203 participants enrolled by outreach workers, 1,186 (98.6%) opted for assisted HIVST, and 1,065 (88.5%) opted for kits that used oral-fluid versus finger-prick blood sample collection. All 7 individuals enrolled through website recruitment (5 MSM and 2 transgender women) opted for unassisted oral-fluid testing.

**TABLE 4.** Participant Uptake of HIV Self-Testing by Key Population Group, Phnom Penh, Cambodia

	Men Who HaveSex With Men		TransgenderWomen		EntertainmentWorkers		Total	
	No. (%)(N=455)	*P* Value	No. (%)(N=325)	*P* Value	No. (%)(N=430)	*P* Value	No. (%)(N=1,210)	*P* Value
Selected HIV testing options		<.001		<.001		<.001		<.001
Assisted HIVST using oral fluid	356 (78.2)		281 (86.5)		413 (96.0)		1,050 (86.8)	
Assisted HIVST using finger prick	89 (19.6)		40 (12.3)		7 (1.6)		136 (11.2)	
Unassisted HIVST using oral fluid	8 (1.8)		4 (1.2)		10 (2.3)		22 (1.8)	
Unassisted HIVST using finger prick	2 (0.4)		0		0		2 (0.2)	
Type of HIVST kit selected		<.001		<.001		<.001		<.001
Oral fluid	364 (80.0)		285 (87.7)		423 (98.4)		1,072 (88.6)	
Finger prick	91 (20.0)		40 (12.3)		7 (1.6)		138 (11.4)	
HIV modalities		<.001		<.001		<.001		<.001
Assisted	445 (97.8)		321 (98.8)		420 (97.7)		1,186 (98.0)	
Unassisted	10 (2.2)		4 (1.2)		10 (2.3)		24 (2.0)	
HIVST kit delivery preference		<.001		<.001		—		<.001
Outreach workers in the community	81 (17.8)		96 (29.5)		430 (100)		607 (50.2	
Community-based organization facility	364 (80.0)		226 (69.5)		0		590 (48.8)	
Courier service	10 (2.2)		3 (0.9)		0		13 (1.1)	

Abbreviation: HIVST, HIV self-testing.

Almost all participants (98%) selected assisted HIVST, and the vast majority (88.6%) opted for the use of an oral-fluid-based test kit.

Table 5 summarizes participants' choice of HIVST kit type, stratified by KP typology, mode of recruitment, and prior testing history. Participants who reported previous testing experience were more likely to opt for oral-fluid versus finger-prick testing (96%), compared to those who reported testing for the first time in the context of the study (86%). Among individuals recruited by outreach workers, a greater proportion of those engaged through face-to-face contact (93%) opted for oral-fluid versus finger-prick testing, compared to those engaged online (61%).

**TABLE 5.** Participant Choice of Test Type by Key Population, Mode of Recruitment, and Prior Testing Experience, Phnom Penh, Cambodia

		Men WhoHave SexWith Men				TransgenderWomen				EntertainmentWorkers				Total	
		Oral-fluid	Finger-prick			Oral-fluid	Finger-prick			Oral-fluid	Finger-prick			Oral-fluid	Finger-prick	
	N	No. (%)	No. (%)	*P* Value	N	No. (%)	No. (%)	*P* Value	N	No. (%)	No. (%)	*P* Value	N	No. (%)	No. (%)	*P* Value
Total participants	455	364 (80.0)	91 (20.0)	<.001	325	285 (87.7)	40 (12.0)	<.001	430	423 (98.4)	7 (1.6)	<.001	1,210	1,072 (88.6)	138 (11.4)	<.001
Mode of recruitment				<.001^a^				=1^a^				=.016^a^				<.001
Outreach workerface-to-face	290	263 (90.7)	27 (9.3)		315	276 (87.6)	39 (12.4)		429	423 (98.6)	6 (1.4)		1,034	962 (93.0)	72 (7.0)	
Outreach workeronline	160	96 (60.0)	64 (40.0)		8	7 (87.5)	1 (12.5)		1	0	1 (100)		169	103 (60.9)	66 (39.1)	
Online advertising	5	5 (100)	0		2	2 (100)	0		0	0	0		7	7 (100)	0	
First-time tester^b^				<.001				<.001				=.604^a^				<.001
Yes	322	240 (74.5)	82 (25.5)		185	150 (81.1)	35 (18.9)		360	353 (98.1)	7(1.9)		867	743 (85.7)	124 (14.3)	
No	125	116 (92.8)	9 (7.2)		137	132 (96.4)	5 (3.6)		69	69 (100)	0		331	317 (95.8)	14 (4.2)	

a Fisher's exact test.

b Excluding a total of 12 observations with "No answer" to the question on HIV testing history: 8 observations for men who have sex with men; 3 for transgender; 1 for entertainment workers.

### HIVST Results and Link to HIV Services

Of the total participants who took an HIVST (N=1,210), 84 (6.9%) received a reactive result (Table 6). The proportion of participants with reactive results varied significantly across KPs: 2.6% EWs, 8.1% MSM, and 11.1% transgender women (*P*<.001). No study participants received invalid HIV testing results.

**TABLE 6.** Participant Test Result by Key Population, Mode of Recruitment, Choice of Testing Option, and Previous Testing History, Phnom Penh, Cambodia

		Men Who HaveSex With Men				TransgenderWomen				EntertainmentWorkers				Total		*P* Value
	N	Reactive No. (%)	Non-reactive No. (%)	*P* Value	N	Reactive No. (%)	Non-reactive No. (%)	*P* Value	N	Reactive No. (%)	Non-reactive No. (%)	*P* Value	N	Reactive No. (%)	Non-reactive No. (%)	
Total participants	455	37 (8.1)	418 (91.9)	<.001	325	36 (11.1)	289 (88.9)	<.001	430	11 (2.6)	419 (97.4)	<.001	1,210	84 (6.9)	1,126 (93.1)	<.001
Mode of recruitment				=.105^a^				=.248^a^				=.974^a^				=.050^a^
Outreach worker face-to-face	290	28 (9.7)	262 (90.3)		315	35 (11.1)	280 (88.9)		429	11 (2.6)	418 (97.4)		1,034	74 (7.2)	960 (92.8)	
Outreach worker online	160	8 (5.0)	152 (95.0)		8	0	8 (100)		1	0	1 (100)		169	8 (4.7)	161 (95.3)	
Online advertising	5	1 (20.0)	4 (80.0)		2	1 (50.0)	1 (50.0)		0	0	0		7	2 (28.6)	5 (71.4)	
Outreach vs. website enrollment				=.347^a^				=.210^a^				-				=.080^a^
Outreach	450	36 (8.0)	414 (92.0)		323	35 (10.8)	288 (89.2)		430	11 (2.6)	419 (97.4)		1,203	82 (6.8)	1,121 (93.2)	
Online advertising	5	1 (20.0)	4 (80.0)		2	1 (50.0)	1 (50.0)		0	0	0		7	2 (28.6)	5 (71.4)	
Choice of HIVST option				=.576^a^				=.062^a^				=1.0^a^				=.229^a^
Assisted	445	36 (8.1)	409 (91.9)		321	34 (10.6)	287 (89.4)		420	11 (2.6)	409 (97.4)		1,186	81 (6.8)	1,105 (93.2)	
Unassisted	10	1 (10.0)	9 (90.0)		4	2 (50.0)	2 (50.0)		10	0	10 (100)		24	3 (12.5)	21 (87.5)	
Choice of HIVST kit type				=.548				=1.0^a^				=1.0^a^				=.881
Oral fluid	364	31 (8.5)	333 (91.5)		285	32 (11.2)	253 (88.8)		423	11 (2.6)	412 (97.4)		1,072	74 (6.9)	998 (93.1)	
Finger prick	91	6 (6.6)	85 (93.4)		40	4 (10.0)	36 (90.0)		7	0	7 (100)		138	10 (7.2)	128 (92.8)	
First-time tester^b^				=.208				=.445				=.225^a^				=.050
Yes	322	22 (6.8)	300 (93.2)		185	18 (9.7)	167 (90.3)		360	11 (3.1)	349 (96.9)		867	51 (5.9)	816 (94.1)	
No	125	13 (10.4)	112 (89.6)		137	17 (12.4)	120 (87.6)		69	0	69 (100)		331	30 (9.1)	301 (90.9)	

Abbreviation: HIVST, HIV self-testing.

a Fisher's exact test.

b Excluding a total of 12 observations with "No answer" to the question on HIV testing history: 8 observations for men who have sex with men; 3 for transgender; 1 for entertainment workers.

When comparing the proportions of participants with a reactive result across the mode of enrollment (outreach versus website), HIVST option chosen (assisted versus unassisted), and HIVST kit type chosen (oral fluid versus finger prick), no statistically significant associations were found overall or by KP typology (Table 6). The overall proportion of participants who received a reactive HIVST result was significantly different between first-time and repeat testers: 5.9% versus 9.1%, respectively (*P*=.050). However, this association was not observed in stratified analyses of these results by specific KP typologies (Table 6).

Among all 84 individuals with reactive HIVST results, 83 (98.8%) received confirmation of their HIV status with the national HIV testing algorithm, and 81 (97.5%) initiated ART during the study period, all at the KP-friendly Chhouk Sar Association Clinic. The 1 individual with reactive results who did not receive confirmatory testing declined multiple attempts by study staff to offer support for confirmatory testing and was ultimately lost to study follow-up.

### Adverse Events and Safety

There were no identified adverse events other than an attempted suicide 2 months before study completion by a transgender woman aged 17 years who was recruited via website and who did not opt for online counseling or assistance. This was immediately reported as a severe adverse event and, in consultation with the Cambodia Ministry of Health, a decision was made to suspend all forms of recruitment that did not incorporate provision of face-to-face counseling for the outstanding duration of the study. The client had registered online, performed the test herself immediately upon delivery, and within 2 hours attempted to hang herself and was found by family and admitted to the hospital. The family contacted the study team members, who were able to visit the hospital to provide support. She was discharged the following day, and study team members assisted her in connecting with a study site for social support, confirmatory HIV testing, and same-day initiation of HIV treatment. As of March 2021, she is healthy and has been on HIV treatment for more than 1 year.

## DISCUSSION

In line with numerous studies in other settings, our findings support the acceptability and feasibility of HIVST among MSM, transgender women, and female EWs in Cambodia.^8^^,^^10^^,^^11^^,^^20^ The opportunity to engage in HIVST through the study attracted the participation of many KP individuals who had not previously accessed HIV testing or other HIV-related services. Current estimates of HIV testing coverage and awareness of one's HIV status among MSM and transgender women in Cambodia are 51.9% and 66.8%, respectively.^21^ Coverage estimates based on program data among sex workers are as high as 100%,^21^ but 83.7% of the EW engaged through this study were first-time testers. All the participants opted for HIVST over referrals to clinic-based HIV testing services, and virtually all participants selected assisted versus unassisted offers of HIVST. Most participants preferred the use of oral-fluid test kits as compared to blood-based kits with finger-prick sample collection. Notably, individuals with prior HIV testing experience—presumably using blood-based samples given historical testing options in Cambodia—were significantly more likely to select oral-fluid testing over blood-based testing. Individuals who were recruited through face-to-face contact were also significantly more likely to opt for oral-fluid-based testing, perhaps indicating that the direct availability of support helped to encourage interest in this previously unfamiliar option.

The opportunity to engage in HIVST through study participation attracted many KP individuals who had not previously accessed HIV testing or other HIV-related services.

The provision of HIVST in this pilot activity resulted in relatively high rates of HIV case detection in each KP group as compared to existing HIV prevalence estimates of 2.3% among sex workers, 4.0% among MSM, and 9.6% among transgender women.^21^ This outcome is noteworthy for 2 reasons: (1) HIV prevalence estimates include individuals who already know their HIV status and (2) Cambodia has documented achievements with respect to national HIV epidemic control targets of diagnosing more than 95% of all PLHIV, expanding treatment to more than 95% of those diagnosed, and achieving HIV viral suppression among more than 95% receiving treatment.^21^ These data suggest that HIVST may be particularly attractive to individuals facing elevated HIV infection risks as well as those who have not previously accessed HIV testing or other services. The number of participants recruited through online advertisements and website registration was small even before the adverse-event-related restrictions imposed on recruitment through this channel midway through study implementation. Nevertheless, these individuals were more than 4 times more likely to receive reactive HIVST results than those recruited through outreach workers. While more than 100 individuals visited the study registration website, the required study-related forms included lengthy consent, eligibility verification procedures, and the requirement that a cell phone number be provided for enrollment appeared to be a barrier to full participation. We anticipate that in routine implementation of HIVST through online ordering, delivery, and pharmacy distribution, there will be a lower threshold to access that will facilitate substantially higher uptake among KPs and individuals who do not identify as a KP member but may be at high risk for HIV infection and would not otherwise access HIV testing.

Individuals who reported prior HIV testing were more likely than first-time testers to receive a reactive result. This suggests that these individuals had a frequency of HIV exposure risk that surpassed those who were testing for the first time and had newly acquired HIV infection since their last HIV test. It is also possible that they already had received a positive test result—which should have rendered them ineligible for study participation—that they chose not to reveal. The 97% ART initiation rate partially rules out the participation of known positive individuals who may have already been on treatment and who were just seeking to confirm their status. All study participants reported HIV exposure risk per the eligibility criteria, and we did not have the means in the survey data to explore more granular behavioral differences or prior testing history. As one of the intended benefits of HIVST is opening preferred access pathways for underserved individuals with high risks, it may be useful to also consider the benefits for individuals who have testing experience but may prefer the con-venience and discreetness associated with HIVST. Our results did find that previous testers were significantly more likely than first-time testers to choose the novel oral-fluid HIVST approach over blood-based testing when given the choice. Taken together, these findings support the value of HIVST as a differentiated service delivery model that is well-positioned to close access gaps among individuals who have not or might not otherwise access services.^22^

These findings support the value of HIVST as a differentiated service delivery model that is well-positioned to close access gaps among individuals who have not or might not otherwise access services.

One of the globally documented challenges associated with HIVST implementation is linkage to confirmatory HIV testing and relevant treatment or prevention services.^23^^,^^24^ We found that all but 1 of the study participants with reactive HIVST results were linked to confirmatory testing and that more than 97% of these successfully initiated HIV treatment. It is possible that routine study-related follow-up, as well as the fact that most participants chose assisted HIVST, helped to improve linkage outcomes in our experience. As Cambodia works to expand access to unassisted HIVST through website-based delivery and other options based on the evidence we encountered of high reactivity rates among individuals who may prefer these modalities, additional attention may be needed to develop solutions to facilitate, support, and confidentially monitor linkages to confirmation, treatment, and prevention. Unassisted strategies offer remarkable opportunities to scale up access to and distribution of HIVST because they are not as dependent on human resources. That said, unassisted strategies may also be especially challenged to overcome a frequently cited priority to maximize HIVST impact—support for and documentation of linkages to confirmatory testing and care.^3^^,^^4^^,^^23^^,^^24^ Some of the common challenges reported from experiences in implementation include supporting and tracking clients, facilitating links to confirmatory testing and care for those clients with reactive test results,^6^^,^^23^^–^^41^ and monitoring and mitigating adverse events.^25^^,^^27^ In practice, several methods have been implemented to track whether clients used HIVST kits, and if they had reactive results, whether they linked themselves to confirmatory testing and care. These include facility register reviews, hotlines, callback cards, follow-up phone calls, financial incentives, and the use of personal identification numbers.^28^^,^^32^^,^^36^^,^^39^

The severe adverse event reported during study implementation was considered a possibility during the process of study design, and had a favorable resolution at least in part due to the preparedness and swift follow-up of the study staff. Because this adverse event resulted in a decision to eliminate the option for participants enrolled in the final 2 months of the study to select unassisted HIVST, there was potential for an impact on the study findings with respect to HIVST preferences. To assess the possible extent of this impact, the study team went back to quantify the proportion of study participants enrolled before the adverse event and the proportion of these who opted for unassisted HIVST. Of the 1,210 participants, 864 (71%) were enrolled before the adverse event. Among the 864, 840 (97.3%) opted for assisted versus unassisted HIVST, a proportion comparable to the 98% of overall participants who selected assisted HIVST for the full duration of the study. The study team found no association between reactive HIV testing results and choice of assisted versus unassisted HIVST when it went back and narrowed this analysis to include the subset of 864 participants enrolled before the adverse event. These additional analyses found that very few participants (2.7%) enrolled before the adverse event selected unassisted HIVST, suggesting that it is unlikely that many of those enrolled following the adverse event would have done so if the option had been available.

The stigma and discrimination that are unfortunately associated with both HIV and with KPs continue to present substantial barriers to both the mental and physical health of individuals with the greatest HIV-related service needs. Reports of mental health issues associated with positive HIV test results are well documented in relevant studies of individuals accessing facility-based HIV services^42^^–^^46^ and based on available evidence the WHO has generally determined that the public health benefits of expanding HIVST outweigh the risks.^1^ The adverse event documented in our study highlights the need for: (1) systematic counseling to identify potentially fragile clients, (2) close follow-up of unassisted HIV testers to screen adverse events and ensure linkages to services, (3) community-based hotlines with a clear referral system to manage adverse events.

Although the high enrollment rate among eligible contacts (99%) may reflect a study strength, the fact that the study sampling frame included a “take-all” sample of KP members likely to have face-to-face or online interactions with partner CBOs served as a noteworthy limitation of our study. This approach potentially constrained the engagement of broader and different segments of these populations who may prefer not to interact with these organizations, or even to self-identify as KP, in turn limiting the representativeness of the sample and the generalizability of the study results. Efforts were made to recruit a broader sample through online advertising but the online study registration requirements were unfortunately cumbersome, involving 7 pages of client inputs. These requirements likely contributed to low rates of completion and served as a barrier to participation among those reached via website. Given the interest we observed in online access to HIVST kits, we anticipate improved implementation uptake in the absence of study-related enrollment procedures.

The study also relied on self-reported data of health behaviors, service experiences, and links to services following reactive testing results. This may have resulted in under-reporting of behavioral risks and over-reporting of service access, associated with social desirability of disclosure. Nevertheless, the study did make use of self-administered electronic surveys to minimize this potential source of bias. The team had concerns that a more rigorous pursuit of linkage data could jeopardize individual privacy and confidentiality.

Following local dissemination of the study findings, the Cambodia Ministry of Health developed and released new standard operating procedures in June 2020 that endorse HIVST through both assisted and unassisted modalities. The new guidelines support the further expansion of HIVST through peer-mediated and community outreach channels and, importantly, provide the policy foundations to expand unassisted HIVST access through online ordering and pharmacy distribution. These online ordering and pharmacy distribution channels may be critical to extending safe and secure HIV testing access during the coronavirus disease (COVID-19) pandemic and beyond. Members of the study team continue to serve as resources to the national HIV program to assist in the development and implementation of more detailed operational plans for HIVST as it is taken to scale in Cambodia.

## CONCLUSIONS

This study contributes to an expanding global evidence base supporting the potential benefits of HIVST scale-up among KPs. When offered the choice, all study participants opted for HIVST over referrals to facility-based testing services, with the vast majority selecting assisted options and a preference for oral-fluid versus blood-based HIV test kits. The study team correctly anticipated that HIVST would attract a sizable proportion of participants with no prior reported HIV testing history but was surprised to find that individuals with a prior history of HIV testing were both more likely to opt for the use of previously unavailable oral-fluid HIVST kits and were more likely to have reactive results. While few individuals engaged through online advertising completed the required website-based study registration procedures, these participants were significantly more likely to have reactive HIVST results, suggesting the potential benefits of online ordering, pharmacy distribution, and other unassisted options to help close outstanding gaps in HIV diagnosis among individuals facing the greatest infection risks.

The high rates of reactivity among study participants support the notion that HIVST is an empowering additional differentiated service delivery option for individuals at significant risk but reticent to test due to perceived stigma or embarrassment. As such, HIVST has the potential to close the remaining and relatively small gap with respect to Cambodia's first 95 target. We found promising evidence of successful linkages of participants with reactive HIVST results to confirmatory testing and treatment but expect that novel confidential solutions to support and foster linkages will be needed to maximize the benefits of unassisted HIVST scale-up in line with new and supportive HIVST policies recently adopted in Cambodia.

## Footnotes

* Female entertainment workers (EWs) are women or girls who exchange sexual services for money or goods, either regularly or occasionally, and irrespective of whether the activity is consciously defined as income generating. EWs typically meet clients in a range of entertainment venues including beer gardens, restaurants, and karaoke and massage parlors.

^†^
Copies of the study-related staff training materials and participant recruitment materials, as well as the Khmer-language HIVST instructions, are available upon request by writing to Sopha Nith: nsopha@fhi360.org.

## Notes

**Peer Reviewed**

**First published online:** June 14, 2022.
